# Supplementary material for: Patterns of smallpox mortality in London, England, over three centuries
Source: PLoS Biol. 2020 Dec 21;18(12):e3000506. doi: 10.1371/journal.pbio.3000506 (PMC7751884; doi:10.1371/journal.pbio.3000506)
Supplement: Attachment — Submitted filename: KrylovaEarn2020_ResponseToReviewers.pdf [file pbio.3000506.s002.pdf]

Responses to Reviewers of PLoS Biology ms PBIOLOGY-D-19-02569R1 “Patterns of smallpox mortality in London, England, over three centuries”

Olga Krylova and David Earn

July 2, 2020

Dear Roli,

Thank you for the reviews you sent to us more than six months ago. We greatly appreciate your patience in giving us such a long time to revise our manuscript given that we are both overwhelmed with work related to advising governments about COVID-19.

We have worked very hard to make major revisions to the manuscript, following the many excellent suggestions and comments of the four reviewers. For convenience, we include below the text of your decision letter and all the reviews. Our responses are in blue.

Sincerely,
David Earn and Olga Krylova

EDITOR’S DECISION LETTER:

Subject: Your PLOS Biology Submission (PBIOLOGY-D-19-02569R1) - [EMID:5e8e28b0901430bf]

Dear David,

Thank you very much for submitting your manuscript ”Patterns of smallpox mortality in London, England, over three centuries” for consideration as a Short Report at PLOS Biology.

Your manuscript has been evaluated by the PLOS Biology editors, an Academic Editor with relevant expertise, and by four independent reviewers.

You'll see that all four reviewers are broadly positive about the study. However, reviewer #2 (a historical demographer) raises some substantial concerns about the historical aspects, including record-keeping practices over the years; please note that her main review is in the Word attachment. In addition, reviewers #3 and #4 ask for you to expand further on your analyses. Please can you attend to all the concerns raised.

In addition, I think I might've been a bit hasty in asking you to compress the Figures to fit our "Short Report" format. Given the overall enthusiasm expressed by reviewers #1, #3 and #4, the Academic Editor and I would be happy for you to re-expand the Figures and text (as needed) and change back to a full Research Article, should you wish.

Thank you. We have taken you up on this and are submitting as a Research Article.

In light of the reviews (below), we will not be able to accept the current version of the manuscript, but we would welcome resubmission of a much-revised version that takes into account the reviewers' comments. We cannot make any decision about publication until we have seen the revised manuscript and your response to the reviewers' comments. Your revised manuscript is also likely to be sent for further evaluation by the reviewers.

Your revisions should address the specific points made by each reviewer. Please submit a file detailing your responses to the editorial requests and a point-by-point response to all of the reviewers' comments that indicates the changes you have made to the manuscript. In addition to a clean copy of the manuscript, please upload a 'track-changes' version of your manuscript that specifies the edits made. This should be uploaded as a "Related" file type. You should also cite any additional relevant literature that has been published since the original submission and mention any additional citations in your response.

We do not use Word, so cannot create the "Track Changes" document that you have requested. We could produce a `latex-diff` document, but the changes are so substantial that it is difficult to see how such a document would be useful to the referees. If you would nevertheless like such a document, please let us know.

Please note while forming your response, if your article is accepted, you may have the opportunity to make the peer review history publicly available. The record will include editor decision letters (with reviews) and your responses to reviewer comments. If eligible, we will contact you to opt in or out.

Before you revise your manuscript, please review the following PLOS policy and formatting requirements checklist PDF: http://journals.plos.org/plosbiology/s/file?id=9411/plos-biology-formatting-checklist.pdf. It is helpful if you format your revision according to our requirements - should your paper subsequently be accepted, this will save time at the acceptance stage.

Please note that as a condition of publication PLOS' data policy (http://journals.plos.

org/plosbiology/s/data-availability) requires that you make available all data used to draw the conclusions arrived at in your manuscript. If you have not already done so, you must include any data used in your manuscript either in appropriate repositories, within the body of the manuscript, or as supporting information (N.B. this includes any numerical values that were used to generate graphs, histograms etc.). For an example see here: http://www.plosbiology.org/article/info%3Adoi%2F10.1371%2Fjournal.pbio.1001908#s5.

For manuscripts submitted on or after 1st July 2019, we require the original, uncropped and minimally adjusted images supporting all blot and gel results reported in an article's figures or Supporting Information files. We will require these files before a manuscript can be accepted so please prepare them now, if you have not already uploaded them. Please carefully read our guidelines for how to prepare and upload this data: https://journals.plos.org/plosbiology/s/figures#loc-blot-and-gel-reporting-requirements.

Upon resubmission, the editors will assess your revision and if the editors and Academic Editor feel that the revised manuscript remains appropriate for the journal, we will send the manuscript for re-review. We aim to consult the same Academic Editor and reviewers for revised manuscripts but may consult others if needed.

We expect to receive your revised manuscript within two months. Please email us (plosbiology@plos.org) to discuss this if you have any questions or concerns, or would like to request an extension. At this stage, your manuscript remains formally under active consideration at our journal; please notify us by email if you do not wish to submit a revision and instead wish to pursue publication elsewhere, so that we may end consideration of the manuscript at PLOS Biology.

When you are ready to submit a revised version of your manuscript, please go to https://www.editorialmanager.com/pbiology/ and log in as an Author. Click the link labelled 'Submissions Needing Revision' where you will find your submission record.

Thank you again for your submission to our journal. We hope that our editorial process has been constructive thus far, and we welcome your feedback at any time. Please don't hesitate to contact us if you have any questions or comments.

Sincerely,

Roli

Roland G Roberts, PhD, Senior Editor PLOS Biology

REVIEWERS' COMMENTS:

Reviewer #1:

[identifies himself as David N. Fisman]

This is a magnificent paper, and highly informative. It is mathematically sophisticated but clearly written and easily understood by non-mathematicians.

Thank you for these kind remarks!

I have 2 minor comments:

1. Unless I'm mistaken, "prodrom" is not a variant spelling of "prodrome". At any rate, prodrome (with e) is the more familiar term.

Thanks. We have removed the section on the natural history of smallpox, following suggestions of other reviewers, so that term no longer appears in the text.

2. Eczema vaccinatum isn't "severe eczema". It represents extensive cutaneous infection in individuals with pre-existing eczema or atopic dermatitis. This can happen with other viruses too (c.f., eczema herpeticum). Would revise terminology.

Thanks. We have changed "severe eczema" to "serious rash" as on the CDC website (which we now cite).

Reviewer #2:

[identifies herself as Romola Davenport]

IMPORTANT: More detailed comments from this reviewer are available in the attached, downloadable Word document.

This paper reports for the first time an analysis of high frequency counts of smallpox burials and deaths in London over almost 300 years. The authors juxtapose their analyses with historical factors, especially relating to the smallpox control measures, that they claim account for the major changes they identify in smallpox mortality matterns in London. The patterns presented are very interesting however the paper's conclusions are not justified by the method used. The method adopted relies on visual comparison of smallpox patterns with an historical timeline. This is unsatisfactory, but would suffice for an exploratory paper that was designed to point the way to further research. However at present the method is used with insufficient rigour with respect to data quality and historical accuracy.

I recommend that the authors make further adjustments to the smallpox burial data to correct for known shortcomings, and rewrite sections of the paper to enhance clarity and

to acknowledge more fully the tenuous nature of some of the claims regarding the effects of smallpox control measures. These recommendations are set out more fully in an attached document.

Thank you for these general comments, and all your more detailed comments to which we reply point-by-point below. We are now very careful not to appear to be claiming to identify any causal connections between control measures and smallpox dynamics, and have made the suggested data adjustments.

This is an interesting paper and I enjoyed reading it. It presents an analysis of weekly counts of burials in London over the period 1664 – 1930 to argue that sequential changes in smallpox control behaviours produced changes in the size and frequency of smallpox epidemics. This conclusion is in contrast to previous analyses of annual burial patterns, where researchers argued that changes in epidemic frequency were a function of changing population size, before the advent of vaccination.

Although the authors use wavelet analysis, their approach is largely illustrative and depends on the juxtaposition of smallpox data with historical events. This is an unsatisfactory approach, but may be sufficient for an initial exploratory analysis (such as a Short Report). However there are two grounds for thinking that the visual patterns and similarities adduced by the authors are unreliable: (1) insufficient attention to the quality of the underlying data, and (2) the rather cavalier treatment of historical evidence. These two issues need to be addressed before publication, and are outlined below.

(1) In a number of cases the authors attribute changes in the patterns of smallpox burials to historical events, when these changes could be more easily explained by changes in the recording of smallpox burials. The London bills of mortality have a number of shortcomings that must be adjusted for before analysis. The most pertinent of these is the progressive tendency from c.1780 for parishes to report the annual total of burials in the last week of the reporting year. The authors note this tendency, but only adjust the burial series for this defect from 1796, the date at which they consider the influence of vaccination to have begun. That is, they use their own pre-defined views of the periodization of smallpox patterns to adjust the underlying data.

Our "Data" section is now much improved and discusses all the issues mentioned above, and others. We have now carefully adjusted for heaping beginning in 1760.

The authors adjusted smallpox burials for heaping in December for the period 1796-1842 (or 1841?), by replacing the excess of burials in the first week of December with the average of adjacent weeks, and then distributing the excess burials evenly over the remaining weeks of the reporting year (page 7). There are two problems with this method. First, burials tended to heap on the last week of the reporting year, however this was not always the first week of December. The last reporting week can be identified from the numbering of weeks in the original returns, and so this number (either 51, 52, or 53) can be used to identify the last week of reporting in each year and to adjust the burials. The authors probably did this but have not made it clear in the text.

As now described in the "Heaping" subsection of our "Data" section, we supplemented automatic detection of heaping with carefully examining every year and making sure no heaping weeks were missed.

The second problem is that the method used to redistribute heaped smallpox burials assumed that these burials were spread evenly throughout he reporting year. This is unlikely. The smallpox burials that were reported in weekly totals showed seasonal patterns. This is because while some parishes became lax in reporting and only reported once a year, others parishes continued to report weekly, and so the remaining variation in weekly totals probably reflected the underlying seasonality of smallpox burials in London. It would therefore make more sense to redistribute the heaped smallpox burials according to the proportional weekly distributions of unheaped smallpox burials in each year.

We agree completely and now redistribute heaped burials according to the proportional weekly distribution of the unheaped burials as you suggest.

The authors' method for redistributing heaped burials in the bills of mortality between 1796 and 1842 (or 1841? See below) may explain the noisiness of the epidemic cycles in the period before 1843, compared with afterwards (Figure 2), when the data source switches from the London bills to the Registrar-General's weekly returns. The authors attribute the noisiness in the data between 1796 and 1840 to the co-existence of variolation and vaccination, before the banning of variolation in 1840. However the noisiness appears to disappear after 1842, suggesting that it is primarily associated with the switch to a much more dependable source of weekly data (Figure 3, middle panel). It may be very difficult to discern any effect of the banning of variolation given its coincidence with a major shift in the quality of the underlying data.

Redistributing the heaped burials more appropriately does not affect the degree of noise in the data, and we have not detected any visually apparent differences. In our current "Results" section, the subsection "Patterns in the normalized time series" now contains the following paragraph:

> "The years from 1770 to 1810 were characterized by stricter regularity of epidemics. This period coincided with more common variolation (the practice gained popularity after the Suttonian innovation of 1768). Beginning around 1810, the data show a dramatic reduction in the amplitude of epidemics, though outbreaks were more frequent and the data are noisier. The apparently declining trend in epidemic severity is temporally associated with the introduction vaccination; however, a causal link would be difficult to establish since this is precisely the period over which the parish registration system collapsed."

(2) The authors have chosen rather arbitrary dates to define historical influences, and this undermines their argument for congruence between historical and epidemiological processes. The Industrial Revolution did not start in 1780, and was not associated with a sudden flood of migrants into London. Other work cited by the authors suggests that adult victims formed a smaller proportion of smallpox victims in London after c.1770. Moreover the argument that

a flood of new susceptibles into London was associated with 'increasingly severe' epidemics (p.11 line 283) is inconsistent with the evidence of a reduction in the (normalised) impact of smallpox in the last quarter of the eighteenth century (Figure 2, last panel and page 15, line 431). The impression of increasingly large individual epidemics may have been caused by the uncorrected heaping of burials on single weeks in December before 1796 (which would produce very large spikes of burials in individual weeks). I would recommend that the reference to the Industrial Revolution be removed, or complemented with a fuller discussion of the effects of changing population size and composition on smallpox patterns.

We now annotate the Industrial Revolution as occuring during 1760–1830 as indicated in Encyclopedia Britannica: "the first Industrial Revolution lasted from the mid-18th century to about 1830."

Our discussion of relevant historical events and previous work on smallpox dynamics in London has been heavily revised. We are careful not to seem to be making causal claims, and we are much more explicit about the hypotheses and inferences that have been drawn previously about a reduction in the mean age at infection around 1770.

Similarly, the effects of vaccination could not have started to manifest in 1796, two years before Jenner published his results. This may seem petty, however the use of dates that are clearly wrong suggests to more historically-minded readers that the authors are not really interested in the influence of historical events and processes, which is unfortunate.

Indeed the authors also give the impression at times that they are imposing their version of events on the data. For example, in discussing variolation on page 8 they state that 'The impact of smallpox did not change until preventative measures were introduced at the beginning of the 18th century' (lines 237-239). However later in discussing their wavelet analyses they note that 'From 1664 until 1700 the dominant period was 3-4 years. Around 1705 it shifted to 2-3 years' (page 15, lines 447-448). The latter description is consistent with the claims of Duncan et al. that population size and growth influenced epidemic patterns, and points to other influences in addition to or more important than variolation practices.

We hope the reviewer will find our revised text, which includes extended discussion of many issues, to be suitable and useful. Again, we have been careful not to claim any causal links, but to highlight issues we believe deserve further attention. We have included an additional graph comparing the inter-epidemic intervals estimated by Duncan *et al* and Cliff *et al* with the spectral peaks we obtained from our wavelet analysis.

Minor issues:

The introductory section provides a rather lengthy and conventional overview of the natural history and global history of smallpox. This could be shortened considerably, and made much more interesting and topical by reference to recent discoveries with respect to the molecular phylogeny of smallpox (e.g. Duggan et al., *Current Biology*, 2016). A key question is whether changes in smallpox control practices exerted evolutionary pressures that led to changes in the properties of circulating strains or to the diversification of smallpox lineages. I would remove the section on 'Types of smallpox' (pp. 4-5) or provide an updated discussion

that also referred to alastrim.

Thank you for these sensible suggestions. We have greatly reduced the length of the introduction, and sharpenned the focus of our review of background (including removing discussion of types and natural history of smallpox). In addition, we now make reference to the Duggan et al study (done at McMaster!) in the “Interpretation” subsection of our current “Discussion”.

The authors tend to elide London and England in their discussions of data, e.g. pages 6 and 7. The Bills of Mortality refer only to London, not England, and were compiled from Anglican registers kept, by law, in individual London parishes. The Registrar-General’s office was not created to remedy the problems of the London bills of mortality (p.7) but to provide a comprehensive system of registration of births and deaths (instead of baptisms and burials) for the country as a whole, and one that included non-Anglicans who were otherwise excluded from the existing requirement for registration of baptism and burials.

We heavily revised the “Data” section and we hope there are no remaining traces of elision of London and England (there are still places where we refer to England rather than London specifically, because we are describing things not specific to London). We now state that the RGO was created to “provide a comprehensive and accurate national registration system of births, marriages and deaths, including causes of death”.

A more minor problem is that the London bills of mortality covered a smaller area than the administrative area of the Registrar-General’s weekly reports. This is not mentioned directly in the paper, although it was one of the reasons for normalisation. Instead the discrepancies between the weekly bills and the weekly returns are attributed solely to reduced accuracy of the bills (Figure 2). However while the bills probably tended to under-report burials within the reporting area, due to laxity of registration and the progress of non-Anglican sects, they also represented a progressively shrinking fraction of the metropolitan population, as a consequence of the expansion of London suburbs outside the area of the bills. Therefore the extrapolation of births in the middle panel of Figure 2 over-states the deficiencies of the bills. As the area included within the bills became built-up, the population, and therefore the numbers of baptisms and burials, would be expected to stabilise, as the figure indicates. Some brief explanation of the shift in administrative area between the bills and the returns would help to resolve this.

We now directly address the shortcomings of the bills, and the different geographical coverage of the RG returns. We no longer extrapolate births.

p.3 lines 50, 53. Replace ‘injection’ with ‘deliberate infection’ with reference to variolation and inoculation.

Done.

p.6 line 157. Replace ‘release of toxins in the blood’ with ‘release of toxins into the blood’.

This section has been removed.

p.6 line 168. Replace ‘Registration of deaths’ with ‘Registration of burials’.

Done.

p.7 line 200. Why were the Registrar-General's weekly returns used from 1842, rather than 1840, when they became available?

When DE's research assistants were in London photographing the original documents, they obtained the LBoM up to 1845 and the RGWR starting in 1842 rather than 1840. This was unfortunate, but should not affect any conclusions of this paper

p.7 line 202. Should the phrase '1796-1842' be '1796-1841'? Were the weekly returns used from 1842?

This part of the text has been revised. As we now clarify, the weekly returns were used from January 1842 onwards.

p.12 line 332. 'of 1871' not italicised.

Done.

p.15 lines 429-30. The normalised measure of smallpox is not a mortality rate, and does not necessarily indicate the trend in *per capita* smallpox mortality. It is a measure of the relative contribution of smallpox to deaths from all causes. Changes in the total death rate would produce changes in the normalised smallpox measure, without any necessary changes in the smallpox death rate (deaths from smallpox per 1,000 population).

We are now careful to refer to the proportion of all-cause mortality.

Reviewer #3:

This article reports the first analyses of a deeply fascinating dataset: the records of smallpox mortality in London from 1664-1930. Digitizing and making publicly available this dataset is a tremendous advance in and of itself, as there are a large number of follow-up studies that will be able to explore different features of this dataset and use it to uncover the influences of demographic, social, and public health change on the dynamics of infectious disease. It is therefore an important contribution, worth publication in a journal with the exposure of PLoS Biology.

Thank you for these kind remarks.

As a Short Report, I realize that the analyses do not necessarily need to be complete. And yet, I was left wanting more from even these preliminary analyses. In particular, the article goes into extensive detail about the history of smallpox in London, but the connection between the statistical analyses and this story is only explored in a very limited fashion. In particular, while Fig. 2 and Fig. 4 both annotate the data with major historical events that might be relevant to smallpox transmission and mortality, the discussion of the connection between any patterns observed in the data and these events in the Results or Discussion

is very limited. For example, on lines 454-462, the authors note several timepoints where seasonality shifts, but those timepoints are not explicitly related back to any of the key events in the history of smallpox. Or, for another example, on lines 422-428, there is more connection between the regularity of epidemics and the history of variolation and vaccination, but there is no discussion of whether the patterns observed in the data (e.g. the shift to very regular epidemics with the introduction of variolation) make sense given the likely effects of control measures on transmission. This carries over into the Discussion as well, which was too brief given all of the potential for a very interesting discussion of a very interesting dataset.

We have substantially expanded and improved our exposition in the "Results" and "Discussion" sections, including much more commentary.

I do think there is plenty of space available to expound on the connection between the statistical analyses and the historical transitions in both London's demography and the control of smallpox, as the historical sections can probably be shortened or eliminated without much loss. In particular, the entire section on "Types of Smallpox" (including Fig. 1) could be removed without loss of content, as none of the information in that section has any bearing on the analysis presented in the paper. Much of that information (e.g., on incubation periods and infectiousness) would be relevant to an paper attempting to fit an epidemiological model to the data, which I assume will happen in a follow-up paper. Similarly, the section, "Smallpox history" could also be drastically shortened: it is included only "to provide context" (line 27), but it is unclear to me how that context is actually helpful, given the detailed discussion of smallpox history in London that follows, especially in the "Annotation of data with historical events" section.

We have followed all these sensible suggestions (including removing the figure and section on types and natural history of smallpox). Indeed, we do plan a followup paper using a mechanistic epidemiological model.

In sum, I think this is a very interesting dataset and analysis, and that some effort to tighten the connection between that analysis and the historical events discussed in the paper would make it a very valuable contribution.

Reviewer #4:

This is a unique and fascinating study on the long-term population dynamics of smallpox. The assembling and curating of such a long record with concurrent relevant events on the history of control is of major value to the field of population dynamics of infectious diseases in general.

Thank you for these kind remarks.

I have however a number of comments intended to clarify the analyses and to strengthen the conclusions. I believe the authors should be able to address these, and to go further in the

interpretation of the results and the kinds of questions this data set, unique in its length, will allow them or others to address in the future, given the results here and what is already known about seasonal SIR dynamics. On this last aspect, I have found the paper a bit thin.

1) The changes in seasonality are an important result. A main conclusion is a shift from summer to winter (line 490 Discussion). The analysis presented in Fig 4b is difficult to 'see'. That is, one can read the caption and text but it is difficult to actually see the described patterns in the figure itself. This is because with so many years compressed in the x axis, one cannot easily follow where the main season is for a given year and how the trends go. I would recommend additional plots to make this sufficiently clear. For example, boxplots representative of the seasonal patterns in selected windows of time would help despite the non-stationarity of the 'average' seasonal cycle.

We experimented with a number of representations of seasonality and converged on what we hope the reviewer will find to be a compelling figure (Fig 4D).

(A smaller comment: the sentence on the zeros not being represented in the caption was confusing. The effect of the detrending needs to be clarified).

Please see the "Seasonality" subsection of the "Methods" section. We hope our explanation of this issue is now clear.

Another important conclusion on the seasonality is in Line 471, where the authors write about changes in the strength of the annual power. I could not see where this comes from. The Fourier power spectrum averages over time, and as far as I could tell, the wavelet spectrum, which doesn't, was applied after filtering the annual variation, so one cannot see there the non-stationarity of the annual power.

Annual variation was not filtered out before constructing the wavelet spectrum. There is, in fact, clear annual power from the beginning of the time series until the early 1800s, as we now mention in the "Seasonality" subsection of the "Discussion" section.

2) Another major result concerns the changes over time of the interannual variability; that is, the changes in the dominant periods of multi-annual cycles. These changes are interpreted as the consequence of control rather than birth rates (and contrasted to the importance assigned to demography in studies of other seasonal infectious diseases). This is where I would have liked to see a clearer exposition of why demography is not an important driver of the changes. From previous influential work on measles and childhood infections (including contributions of one of the authors, David Earn), we know that birth rates, transmission rates, and vaccination coverage, can have equivalent/related effects on seasonal SIR dynamics. How do the patterns/trends here relate to that previous knowledge and how can one infer the importance or dominance of control over demography?

We did not mean to imply that secular changes in birth rates are not important. We have tried to be clearer about this, and emphasize that many factors could have contributed to changing transmission rates as well. Our purpose in this paper is to describe the patterns and set the stage for future analyses based on mechanistic mathematical models, as we indicate

at the end of the “Discussion” section.

3) Are there ways to look at the changes in the intensity of the annual cycle in relation to the importance of other, longer, cycles? We know from earlier theory how the spectrum of seasonal SIR dynamics should change with demography and transmission intensity. Are there concurrent patterns in how the power is distributed over different periods that would relate to that theory and allow interpretation?

There certainly are ways to explore these things using mechanistic models, and it seems the reviewer is familiar with some of the second author’s related work on other infectious diseases. The first author did some preliminary work on this in her PhD thesis, and in the meantime DE has worked on improving methods to estimate seasonal variation of contact rates in these long time series, and refine the methodology in several other respects. We do hope to complete a paper on mechanistic analyses of the data presented here before too long.

4) Similarly, a major contribution of this paper will be to make this remarkable data set available to the community. The analyses in the paper itself are largely descriptive of trends in the seasonal and interannual variability and in relation to the timing of control efforts. It would be valuable to go further and based on these patterns, provide some major directions for what these data will allow going forward, in particular from their analyses with process-based models and related hypothesis about smallpox and seasonal SIR dynamics in general.

We appreciate this suggestion and have now included a subsection “Explaining transitions in smallpox dynamics” in our “Discussion” section. We have tried to articulate some of the challenges that must be overcome when following the exciting path that the referee seems to have in mind.
