# Supplementary material for: Patterns of smallpox mortality in London, England, over three centuries
Source: PLoS Biol. 2020 Dec 21;18(12):e3000506. doi: 10.1371/journal.pbio.3000506 (PMC7751884; doi:10.1371/journal.pbio.3000506)
Supplement: Attachment — Submitted filename: KrylovaEarn2020_Response2.pdf [file pbio.3000506.s003.pdf]

Responses to Reviewers of PLoS Biology ms PBIOLOGY-D-19-02569R2 "Patterns of smallpox mortality in London, England, over three centuries"

Olga Krylova and David Earn

September 2, 2020

Dear Roli,

Thank you for the second round of reviews of our paper.

We have made the requested revisions and respond point-by-point to the reviewers' comments below. For convenience, we include below the text of your decision letter and all the reviews. Our responses are in blue.

Sincerely,
David Earn and Olga Krylova

EDITOR'S DECISION LETTER:

```
Date: 10 Aug 2020 09:01:07 -0400
Subject: Your PLOS Biology Submission (PBIOLOGY-D-19-02569R2) - [EMID:80236b3b94569ef1]
```

Dear Dr Earn,

Thank you for submitting your revised Research Article entitled "Patterns of smallpox mortality in London, England, over three centuries" for publication in PLOS Biology. I have

now obtained advice from three of the original reviewers and have discussed their comments with the Academic Editor.

Based on the reviews, we will probably accept this manuscript for publication, assuming that you will modify the manuscript to address the remaining points raised by the reviewers. IMPORTANT: The article type still seems to be "Short Report"; please change it to "Research Article" when re-submitting. Please also make sure to address the Data Policy-related requests noted at the end of this email.

Roli Roberts e-mailed on 2 Sep 2020 to say that the journal will take care of the change to “Research Article”.

We expect to receive your revised manuscript within two weeks. Your revisions should address the specific points made by each reviewer. In addition to the remaining revisions and before we will be able to formally accept your manuscript and consider it "in press", we also need to ensure that your article conforms to our guidelines. A member of our team will be in touch shortly with a set of requests. As we can't proceed until these requirements are met, your swift response will help prevent delays to publication.

Copyediting
Upon acceptance of your article, your final files will be copyedited and typeset into the final PDF. While you will have an opportunity to review these files as proofs, PLOS will only permit corrections to spelling or significant scientific errors. Therefore, please take this final revision time to assess and make any remaining major changes to your manuscript.

NOTE: If Supporting Information files are included with your article, note that these are not copyedited and will be published as they are submitted. Please ensure that these files are legible and of high quality (at least 300 dpi) in an easily accessible file format. For this reason, please be aware that any references listed in an SI file will not be indexed. For more information, see our Supporting Information guidelines: `https://journals.plos.org/plosbiology/s/supporting-information`

Published Peer Review History
Please note that you may have the opportunity to make the peer review history publicly available. The record will include editor decision letters (with reviews) and your responses to reviewer comments. If eligible, we will contact you to opt in or out. Please see here for more details: `https://blogs.plos.org/plos/2019/05/plos-journals-now-open-for-published-peer-revie`[illegible]

Early Version
Please note that an uncorrected proof of your manuscript will be published online ahead of the final version, unless you opted out when submitting your manuscript. If, for any reason, you do not want an earlier version of your manuscript published online, uncheck the box. Should you, your institution's press office or the journal office choose to press release your paper, you will automatically be opted out of early publication. We ask that you notify us as soon as possible if you or your institution is planning to press release the article.

Protocols deposition

To enhance the reproducibility of your results, we recommend that if applicable you deposit your laboratory protocols in protocols.io, where a protocol can be assigned its own identifier (DOI) such that it can be cited independently in the future. For instructions see: https://journals.plos.org/plosbiology/s/submission-guidelines#loc-materials-and-methods

Submitting Your Revision
To submit your revision, please go to https://www.editorialmanager.com/pbiology/ and log in as an Author. Click the link labelled 'Submissions Needing Revision' to find your submission record. Your revised submission must include a cover letter, a Response to Reviewers file that provides a detailed response to the reviewers' comments (if applicable), and a track-changes file indicating any changes that you have made to the manuscript.

Please do not hesitate to contact me should you have any questions.

Sincerely,

Roli Roberts

Roland G Roberts, PhD,
Senior Editor,
rroberts@plos.org,
PLOS Biology

DATA POLICY:

You may be aware of the PLOS Data Policy, which requires that all data be made available without restriction: http://journals.plos.org/plosbiology/s/data-availability. For more information, please also see this editorial: http://dx.doi.org/10.1371/journal.pbio.1001797

We note that your raw data are deposited in http://iidda.mcmaster.ca - however, we strongly prefer more stable, non-institutional repositories (e.g. Dryad, Figshare, Github), and request that you make such provision for depositing your data and code. At the moment http://iidda.mcmaster.ca is giving a timeout error, which gives us further for the long-term availability of this important dataset.

We understand your concerns and we have created a github repository (https://github.com/davidearn/London_smallpox) that includes all the data and all the R scripts that create our figures. The IIDDA web site should have been functioning by now, but the COVID-19 pandemic has caused many delays. Our plan is for the URL that times out for you at the moment to eventually point to a very stable CKAN link. We will therefore continue to state in the paper that the data are available at IIDDA, as well as at the new github repo. If you prefer to have the data available, in addition, on the PLoS website as a supplementary `.zip` file, please let us know.

In addition, we ask that all individual quantitative observations that underlie the data summarized in the figures and results of your paper be made available in one of the following forms:

1) Supplementary files (e.g., excel). Please ensure that all data files are uploaded as 'Supporting Information' and are invariably referred to (in the manuscript, figure legends, and the Description field when uploading your files) using the following format verbatim: `S1 Data`, `S2 Data`, etc. Multiple panels of a single or even several figures can be included as multiple sheets in one excel file that is saved using exactly the following convention: `S1_Data.xlsx` (using an underscore).

2) Deposition in a publicly available repository. Please also provide the accession code or a reviewer link so that we may view your data before publication.

See https://github.com/davidearn/London_smallpox.

Regardless of the method selected, please ensure that you provide the individual numerical values that underlie the summary data displayed in the following figure panels as they are essential for readers to assess your analysis and to reproduce it: Figs 1, 2, 3, 4, 5, 6, S1, S2. NOTE: the numerical data provided should include all replicates AND the way in which the plotted mean and errors were derived (it should not present only the mean/average values).

Please also ensure that figure legends in your manuscript include information on where the underlying data can be found, and ensure your supplemental data file/s has a legend.

We have added the following statement to the caption for each figure (other than Fig 1, which is just a photograph). "The data and R script required to reproduce this figure are available at https://github.com/davidearn/London_smallpox."

Please ensure that your Data Statement in the submission system accurately describes where your data can be found.

REVIEWERS' COMMENTS:

Reviewer #2: [identifies herself as Romola Davenport]

The authors have done a great job in revising the paper. It is much clearer and more tightly written, and presents a very impressive integration of historical and epidemiological literatures. I think it will make a great addition to current debates over the recent evolution of smallpox.

Thanks very much!

I have only minor comments that should be addressed before publication.

1. The authors are now perhaps too reticent in attributing causation, especially with respect to vaccination (page 14/33, lines 431-6). The decline in smallpox deaths with the introduction of vaccination in the early nineteenth century is very marked in both raw and normalised burials. This phenomenon was observed in other cities and states that adopted vaccination, and coincided with a marked decline in all-cause mortality (so the reduction in normalised smallpox burials is likely to underestimate the fall in smallpox mortality).

We have revised the sentence in question, which now reads "The declining trend in epidemic severity is temporally associated with the introduction of vaccination; unfortunately, this was precisely the period over which the parish registration system collapsed, increasing the difficulty of estimating the true impact of vaccination in the early vaccine era."

2. The term 'mortality' usually refers to mortality *rates*, that is, deaths per population at risk. The authors should distinguish clearly when they are talking about counts of deaths or normalised deaths, to avoid confusion. Figure 1 top panel should be labelled as smallpox deaths, not mortality, and the y-axis should read 'weekly smallpox deaths'. The y-axis of Figure 2 should also be labelled 'weekly all-cause deaths'.

We have made the suggested changes to the figure labels.

3. A slightly larger comment: Why were normalised deaths used to study seasonal patterns? Seasonal patterns in raw deaths should be largely unaffected by longer-term changes in reporting units or under-registration (for the same reasons that normalised deaths are preferable for other purposes). The use of raw deaths to study seasonality would avoid the potential distortions caused by other seasonal patterns of mortality. For example, scarlet fever emerged as a major cause of death in London in the 1930s, with a marked autumnal pattern. This could have reduced the proportion of all deaths due to smallpox in the autumn, regardless of the underlying seasonal pattern of smallpox mortality in this period. Other important causes of death also showed seasonal patterns, and some of these changed over the period of the study (including measles). The authors should acknowledge this potential problem, if they prefer to use normalised burials and deaths.

We believe that the referee has mistakenly inferred that we normalized by weekly all-cause deaths. Had we done so, we would agree that this would interfere with our ability to detect seasonal patterns in smallpox. Indeed, in the extreme that most deaths were attributed to smallpox, dividing by all-cause deaths would remove the seasonality altogether.

In fact, as we explain in the Normalization subsection of the Methods section, we normalized smallpox deaths by the long term trend in all-cause deaths, which has no seasonality. Smallpox deaths are therefore scaled conveniently without affecting seasonal patterns.

4. Table 1 (appendix B): the labels for the third and fourth columns appear to be transposed.

We have re-ordered the columns.

5. page 3/33 line 51: another important element in the eradication of smallpox was the

relatively low infectivity of smallpox.

We have added “relatively low infectivity” to the list.

6. page 3/33 line 60: insert ’and only for a few towns’ between ’until later’ and ’Bills of Mortality’.

Done – thanks.

7. page 4/33, line 81: perhaps replace ’exists’ with ’survives’ (to avoid the impression that patchy series necessarily imply gaps in the production of weekly bills as opposed to survival).

Done – thanks.

8. page10/33, line 256-7: perhaps add ’need for periodic revaccination’ to the list of impediments to vaccine uptake. There was some resurgence of smallpox, and a rise in average age of victims, in the 1820s and 1830s that may have been associated with the waning of vaccine-derived immunity in birth cohorts in which vaccination was very common.

(The correct line reference is 266–7.)

Done – thanks. We have said “waning immunity (hence a need for periodic revaccination)”.

9. Typographical errors: abstract line 1, ’devastated’ for ’devasted’; page 14/33, line 427: ’and’ for ’an’; page 14/35, line 434: insert ’of’ after ’introduction’.

Repaired – thanks for catching these.

Reviewer #3:

I appreciate the revisions that went into this manuscript, and I think it is very close to publication-ready at PLoS Biology. I remain convinced that the data, by itself, is incredibly valuable, and the extended analysis presented here makes this paper a more meaningful contribution as well. I have only a few comments that should be straightforward to address.

I feel that the importance (the ”so what” question) of the analyses presented here needs to be better articulated in the Introduction and Discussion sections. Right now the value is implicit throughout the manuscript, and nowhere do the authors state clearly what they can learn from the statistical analysis of this data, and how will what they learn from these analyses be useful, both for understanding infectious diseases more generally and also for the next phase of work on smallpox specifically (e.g., building mechanistic models).

The end of the Introduction now reads:

> Our statistical descriptions of the weekly smallpox data will help sharpen and quantify research questions concerning the mechanistic origin of changes in the temporal patterns of epidemics [11-14]. In addition, we present a timeline of ma-

jor historical events that occurred during the epoch we have studied. Overlaying the historical timeline with smallpox mortality and prevention patterns provides an illuminating view of three centuries of smallpox history.

In the Discussion, we have added this sentence in the subsection "Explaining transitions in smallpox dynamics":

> Our spectral and seasonal analyses (Figs 5-7) quantify transitions in smallpox dynamics that should be possible to explain using mechanistic mathematical models.

On the description of the handling of the heaped data, you might note that you considered other ways of handling the heaped data (e.g., the way it was handled in the first version of the manuscript) and that this did not have any effect on the conclusions drawn here.

The final bullet point in the description of heaping now reads:

> We calculated the difference between original heaped count and the replaced value (the "excess" due to heaping), and redistributed this number of smallpox deaths in proportion to reported smallpox throughout the year (so the adjusted counts have non-integer values). This redistribution ensured that the original and revised time series contained the same annual numbers of smallpox deaths. (We separately considered redistributing the excess uniformly throughout the year, and did not detect any differences in our results.)

The rationale for identifying the "Intervention uptake levels" in Fig. 1 is never made clear. Why, for example, does the assumed uptake level go from "very low" to "low" in 1728? Why does it go from "low" to "moderate" in 1740, if the first charitable variolation hospital didn't open until 1746? Etc. I realize that this doesn't impact the analyses presented here (because you are not seeking to draw any quantitative conclusions between the dynamics and the level of intervention uptake), but I still think it would be useful to provide some justification.

As we now clarify in the paper, we assume that the level during the period 1728–1740 was between the known very low level before 1728 and the known higher level after 1740. We now provide several references to support our indication of an increase to "Moderate" variolation uptake levels around 1740. For example, Tucker (2002) states "variolation became popular in England by the 1740s". The changes after 1768 are supported by the Razell references that we cite in the main text when referring to the period of increase in popularity of variolation. To emphasize that we are doing the best we can with qualitative information, we now state:

> From these qualitative descriptions, it seems likely that uptake of variolation increased after 1768 and reached a maximum during 1790–1808 [3,61] (annotated in Fig 3).

You do not justify the use of square-root transformation in the spectral analysis section. Also, in this section it would be useful to explain to a reader who has limited exposure to time series analysis what is gained by carrying out both the power spectrum analysis and the wavelet analysis.

We have expanded the introductory paragraph in the "Spectral analysis" section, which now reads:

> We used spectral analyses to identify the strongest periodicities in the smallpox time series, both globally (with a traditional Fourier analysis) and locally (via wavelet analysis). Before computing spectra, we normalized and square-root transformed the data in order to reduce variation in amplitude without affecting periodicities [74,75].

You are missing an "of" on line 434 between "introduction" and "vaccination."

Done – thanks – also mentioned by Reviewer #2.

There is some inconsistency in how Fig. 4B is discussed in the Results and Discussion. In the Results, the power of the annual period is not discussed - you only mention periods at 2, 3, and 5 years. But in the Discussion (lines 465-466), you say that "the wavelet spectrum in Fig. 4B shows a peak at one year," a finding that is not very apparent in Fig. 4B (at least to me).

Thank you. Not mentioning the one-year period in the Results was an oversight. We have added the following sentence in the Results:

> A relatively weak spectral peak at one year can be seen over much of the time series before 1820, though its magnitude is below the threshold for drawing a black peak line except for the decade 1798–1808.

The discussion of possible viral evolution (lines 551-559) could reference some of the theoretical work by Sylvain Gandon and Troy Day on how vaccination is expected to drive pathogen evolution (especially the reference on line 557-559 that suggests the potential for variolation to be "leaky"). E.g., Gandon, S., Mackinnon, M. J., Nee, S., & Read, A. F. (2001). Imperfect vaccines and the evolution of pathogen virulence. Nature, 414(6865), 751-756. Gandon, S., & Day, T. (2007). The evolutionary epidemiology of vaccination. Journal of the Royal Society Interface, 4(16), 803-817.

Thanks – we have now cited these papers.

To help foreshadow the future work you anticipate in response to these data and analyses, you might say a bit more about *how* the studies of measles and other childhood infections were able to explain dynamical transitions evident in the data (liens 583-588).

This comment refers to the "Explaining transitions in smallpox dynamics" section in the Discussion. The paragraph immediately following the cited lines addresses this, and we

are not sure what additional commentary the referee was hoping for. We have added the following sentence at the end of the paragraph in question:

> Our spectral and seasonal analyses (Figs 5–7) quantify transitions in smallpox dynamics that should be possible to explain using mechanistic mathematical models [64,78,97,98].

You are missing an "in" on line 610 between "patterns" and "infectious."

Repaired – thanks for catching this.

Reviewer #4:

The authors have done a great job at clarifying my questions and addressing my concerns in the revision. The revised presentation on the changes in seasonality and on the interannual variation is now very clear. I also appreciated the discussion on the directions these data and patterns open up for future research.

Thank you!

In compliance with data protection regulations, you may request that we remove your personal registration details at any time. (Use the following URL: https://www.editorialmanager.com/pbiology/login.asp?a=r). Please contact the publication office if you have any questions.
