# Supplementary material for: Patterns of smallpox mortality in London, England, over three centuries
Source: PLoS Biol. 2020 Dec 21;18(12):e3000506. doi: 10.1371/journal.pbio.3000506 (PMC7751884; doi:10.1371/journal.pbio.3000506)
Supplement: Attachment — Submitted filename: krylova and earn.docx [file pbio.3000506.s001.docx]

This is an interesting paper and I enjoyed reading it. It presents an analysis of weekly counts of burials in London over the period 1664 – 1930 to argue that sequential changes in smallpox control behaviours produced changes in the size and frequency of smallpox epidemics. This conclusion is in contrast to previous analyses of annual burial patterns, where researchers argued that changes in epidemic frequency were a function of changing population size, before the advent of vaccination.

Although the authors use wavelet analysis, their approach is largely illustrative and depends on the juxtaposition of smallpox data with historical events. This is an unsatisfactory approach, but may be sufficient for an initial exploratory analysis (such as a Short Report). However there are two grounds for thinking that the visual patterns and similarities adduced by the authors are unreliable: (1) insufficient attention to the quality of the underlying data, and (2) the rather cavalier treatment of historical evidence. These two issues need to be addressed before publication, and are outlined below.

(1) In a number of cases the authors attribute changes in the patterns of smallpox burials to historical events, when these changes could be more easily explained by changes in the recording of smallpox burials. The London bills of mortality have a number of shortcomings that must be adjusted for before analysis. The most pertinent of these is the progressive tendency from c.1780 for parishes to report the annual total of burials in the last week of the reporting year. The authors note this tendency, but only adjust the burial series for this defect from 1796, the date at which they consider the influence of vaccination to have begun. That is, they use their own pre-defined views of the periodization of smallpox patterns to adjust the underlying data.

The authors adjusted smallpox burials for heaping in December for the period 1796-1842 (or 1841?), by replacing the excess of burials in the first week of December with the average of adjacent weeks, and then distributing the excess burials evenly over the remaining weeks of the reporting year (page 7). There are two problems with this method. First, burials tended to heap on the last week of the reporting year, however this was not always the first week of December. The last reporting week can be identified from the numbering of weeks in the original returns, and so this number (either 51, 52, or 53) can be used to identify the last week of reporting in each year and to adjust the burials. The authors probably did this but have not made it clear in the text.

The second problem is that the method used to redistribute heaped smallpox burials assumed that these burials were spread evenly throughout he reporting year. This is unlikely. The smallpox burials that were reported in weekly totals showed seasonal patterns. This is because while some parishes became lax in reporting and only reported once a year, others parishes continued to report weekly, and so the remaining variation in weekly totals probably reflected the underlying seasonality of smallpox burials in London. It would therefore make more sense to redistribute the heaped smallpox burials according to the proportional weekly distributions of unheaped smallpox burials in each year.

The authors’ method for redistributing heaped burials in the bills of mortality between 1796 and 1842 (or 1841? See below) may explain the noisiness of the epidemic cycles in the period before 1843, compared with afterwards (Figure 2), when the data source switches from the London bills to the Registrar-General’s weekly returns. The authors attribute the noisiness in the data between 1796 and 1840 to the co-existence of variolation and vaccination, before the banning of variolation in 1840. However the noisiness appears to disappear after 1842, suggesting that it is primarily associated with the switch to a much more dependable source of weekly data (Figure 3, middle panel). It may be very difficult to discern any effect of the banning of variolation given its coincidence with a major shift in the quality of the underlying data.

(2) The authors have chosen rather arbitrary dates to define historical influences, and this undermines their argument for congruence between historical and epidemiological processes. The Industrial Revolution did not start in 1780, and was not associated with a sudden flood of migrants into London. Other work cited by the authors suggests that adult victims formed a smaller proportion of smallpox victims in London after c.1770. Moreover the argument that a flood of new susceptibles into London was associated with ‘increasingly severe’ epidemics (p.11 line 283) is inconsistent with the evidence of a reduction in the (normalised) impact of smallpox in the last quarter of the eighteenth century (Figure 2, last panel and page 15, line 431). The impression of increasingly large individual epidemics may have been caused by the uncorrected heaping of burials on single weeks in December before 1796 (which would produce very large spikes of burials in individual weeks). I would recommend that the reference to the Industrial Revolution be removed, or complemented with a fuller discussion of the effects of changing population size and composition on smallpox patterns.

Similarly, the effects of vaccination could not have started to manifest in 1796, two years before Jenner published his results. This may seem petty, however the use of dates that are clearly wrong suggests to more historically-minded readers that the authors are not really interested in the influence of historical events and processes, which is unfortunate.

Indeed the authors also give the impression at times that they are imposing their version of events on the data. For example, in discussing variolation on page 8 they state that ‘The impact of smallpox did not change until preventative measures were introduced at the beginning of the 18^th^ century’ (lines 237-239). However later in discussing their wavelet analyses they note that ‘From 1664 until 1700 the dominant period was 3-4 years. Around 1705 it shifted to 2-3 years’ (page 15, lines 447-448). The latter description is consistent with the claims of Duncan et al. that population size and growth influenced epidemic patterns, and points to other influences in addition to or more important than variolation practices.

Minor issues:

The introductory section provides a rather lengthy and conventional overview of the natural history and global history of smallpox. This could be shortened considerably, and made much more interesting and topical by reference to recent discoveries with respect to the molecular phylogeny of smallpox (e.g. Duggan et al., *Current Biology*, 2016). A key question is whether changes in smallpox control practices exerted evolutionary pressures that led to changes in the properties of circulating strains or to the diversification of smallpox lineages. I would remove the section on ‘Types of smallpox’ (pp. 4-5) or provide an updated discussion that also referred to alastrim.

The authors tend to elide London and England in their discussions of data, e.g. pages 6 and 7. The Bills of Mortality refer only to London, not England, and were compiled from Anglican registers kept, by law, in individual London parishes. The Registrar-General’s office was not created to remedy the problems of the London bills of mortality (p.7) but to provide a comprehensive system of registration of births and deaths (instead of baptisms and burials) for the country as a whole, and one that included non-Anglicans who were otherwise excluded from the existing requirement for registration of baptism and burials.

A more minor problem is that the London bills of mortality covered a smaller area than the administrative area of the Registrar-General’s weekly reports. This is not mentioned directly in the paper, although it was one of the reasons for normalisation. Instead the discrepancies between the weekly bills and the weekly returns are attributed solely to reduced accuracy of the bills (Figure 2). However while the bills probably tended to under-report burials within the reporting area, due to laxity of registration and the progress of non-Anglican sects, they also represented a progressively shrinking fraction of the metropolitan population, as a consequence of the expansion of London suburbs outside the area of the bills. Therefore the extrapolation of births in the middle panel of Figure 2 over-states the deficiencies of the bills. As the area included within the bills became built-up, the population, and therefore the numbers of baptisms and burials, would be expected to stabilise, as the figure indicates. Some brief explanation of the shift in administrative area between the bills and the returns would help to resolve this.

p. 3 lines 50, 53. Replace ‘injection’ with ‘deliberate infection’ with reference to variolation and inoculation.

p.6 line 157. Replace ‘release of toxins in the blood’ with ‘release of toxins into the blood’.

p. 6 line 168. Replace ‘Registration of deaths’ with ‘Registration of burials’.

p.7 line 200. Why were the Registrar-General’s weekly returns used from 1842, rather than 1840, when they became available?

p.7 line 202. Should the phrase ‘1796-1842’ be ‘1796-1841’? Were the weekly returns used from 1842?

p. 12 line 332. ‘of 1871’ not italicised.

p.15 lines 429-30. The normalised measure of smallpox is not a mortality rate, and does not necessarily indicate the trend in *per capita* smallpox mortality. It is a measure of the relative contribution of smallpox to deaths from all causes. Changes in the total death rate would produce changes in the normalised smallpox measure, without any necessary changes in the smallpox death rate (deaths from smallpox per 1,000 population).
